# Magnetic nanoparticle density mapping from the magnetically induced displacement data: a simulation study

**DOI:** 10.1186/1475-925X-11-11

**Published:** 2012-03-07

**Authors:** ABM Aowlad Hossain, MH Cho, SY Lee

**Affiliations:** 1 Department of Biomedical Engineering, Kyung Hee University, Yongin-si, Gyeonggi-do, Republic of Korea

## Abstract

**Background:**

Magnetic nanoparticles are gaining great roles in biomedical applications as targeted drug delivery agents or targeted imaging contrast agents. In the magnetic nanoparticle applications, quantification of the nanoparticle density deposited in a specified region is of great importance for evaluating the delivery of the drugs or the contrast agents to the targeted tissues. We introduce a method for estimating the nanoparticle density from the displacement of tissues caused by the external magnetic field.

**Methods:**

We can exert magnetic force to the magnetic nanoparticles residing in a living subject by applying magnetic gradient field to them. The nanoparticles under the external magnetic field then exert force to the nearby tissues causing displacement of the tissues. The displacement field induced by the nanoparticles under the external magnetic field is governed by the Navier's equation. We use an approximation method to get the inverse solution of the Navier's equation which represents the magnetic nanoparticle density map when the magnetic nanoparticles are mechanically coupled with the surrounding tissues. To produce the external magnetic field inside a living subject, we propose a coil configuration, the Helmholtz and Maxwell coil pair, that is capable of generating uniform magnetic gradient field. We have estimated the coil currents that can induce measurable displacement in soft tissues through finite element method (FEM) analysis.

**Results:**

From the displacement data obtained from FEM analysis of a soft-tissue-mimicking phantom, we have calculated nanoparticle density maps. We obtained the magnetic nanoparticle density maps by approximating the Navier's equation to the Laplacian of the displacement field. The calculated density maps match well to the original density maps, but with some halo artifacts around the high density area. To induce measurable displacement in the living tissues with the proposed coil configuration, we need to apply the coil currents as big as 10^4^A.

**Conclusions:**

We can obtain magnetic nanoparticle maps from the magnetically induced displacement data by approximating the Navier's equation under the assumption of uniform-gradient of the external magnetic field. However, developing a coil driving system with the capacity of up to 10^4^A should be a great technical challenge.

## Background

Molecular imaging is known to be very powerful in early detection of cancers since molecular images show information about molecular or cellular level activities in a living body with much higher contrast of cancer tissues than conventional diagnostic images [1,2]. In molecular imaging, securing high contrast of the targeted molecules or cells against the background tissues is crucial. Nuclear imaging devices like positron emission tomography (PET) or single photon emission computed tomography (SPECT) utilize radio-pharmaceuticals that tend to combine with targeted molecules or cells. Nuclear imaging devices have very high sensitivity and contrast, but they suffer from toxicity of the radio-pharmaceuticals and long scan time [2]. Optical imaging devices, particularly fluorescence and bioluminescence imaging devices, are believed to be most versatile for molecular and cellular imaging since fluorescent and bioluminescent probes have unparalleled sensitivity and specificity in detecting biochemical activities [2]. Optical imaging devices, however, suffer from very limited imaging depth, which is particularly problematic in human imaging. Magnetic resonance imaging (MRI) uses magnetic nanoparticles for molecular or cellular imaging. In MRI, the main magnetic field, usually an order of Tesla, magnetizes the nanoparticles injected to the living subject and the nanoparticles make detectable interference pattern in resulting magnetic resonance images [2]. However, molecular MRI suffers from long scan time due to its low sensitivity.

Recently, it has been reported that ultrasound imaging can be used for molecular or cellular imaging with aids of magnetic nanoparticles [3]. If we apply strong magnetic field to a living body into which magnetic nanoparticles are administered, the magnetic field produce magnetic propulsion force that can induce spatial displacement of the magnetic nanoparticles and surrounding tissues. If we measure the displacement using ultrasound imaging techniques, we can obtain information about migration of the magnetic nanoparticles in the living body. Feasibility of ultrasound molecular imaging using magnetic nanoparticles is suggested in a recent *in vitro *study of a mouse liver [4].

Quantification and visualization of molecular probe density at a region of interest is of crucial importance to understand the molecular or cellular activities in the living body. The previous studies on molecular ultrasound imaging with magnetic nanoparticles have been limited to discriminating existence of the nanoparticles at a region of interest. In this paper, we propose a method for quantitative magnetic-nanoparticle-density mapping from the magnetically induced displacement data. We also propose a magnetic coil configuration to realize the proposed method.

## Methods

### Application of magnetic force to the magnetic particles to induce displacement

In molecular ultrasound imaging of tissues with magnetic particles in them, quantification of magnetic particle distribution in the region of interest is of great interest. For the quantification, it is most desirable to make the pixel intensity proportional to the particle density. To sensitize magnetic particles by ultrasound, moving the magnetic particles by external magnetic field is thought to be most efficient. We can detect the magnetic particle movement either by measuring Doppler frequency shift or tissue displacements at the region containing the magnetic particles. The magnetic force **F **[N] exerted on a magnetic dipole moment **m **[A·m^2^] by the external magnetic field **B **[Tesla] is given as [5-8]:

(1) F=(m⋅∇)B

When both **m **and **B **point to the same direction, for example **m **= *m_z_***a**_z_, **B **= *B_z_***a**_z_, in a region of interest as is often the case for magnetic propulsion of magnetic particles [7], the magnetic force becomes $F_{z}=m_{z}\frac{\partial B_{z}}{\partial z}$. This means that we need magnetic field gradient to apply magnetic force on the magnetic moment. Usually magnetic nanoparticles show superparamagnetism [9]. Superparamagnetic nanoparticles are magnetized by the external magnetic field, and they have little hysteresis. If we apply spatially uniform magnetic field **H**_0 _= *H*_0_**a***_z _*[A/m] to the nanoparticle containing region, the magnetic moment of a nanoparticle will be **m **= χ**H_o _**[A·m^2^] where χ is the susceptibility of the magnetic nanoparticle. At a pixel of volume *V *[m^3^] containing multiple particles, we can define the effective magnetization of the pixel as $M=\underset{k}{\sum }m_{k}/V$ [A/m] where **m***_k _*is the magnetic moment of the *k*-th particle. If we apply the magnetic gradient field along with the uniform field **H_o_**, the magnetic force exerted on the pixel of interest will be *F_z _*= *VM_z_*(∂*B_z_*/∂z). Therefore, to make the magnetic force map be proportional to the magnetic particle density map, it is necessary to apply uniform magnetic field gradient over the region of interest.

### Magnetic nanoparticle density calculation from the displacement data

When a deformable object is subject to an external force, the external force makes a displacement field in the object deforming it. Development of the displacement field inside the object is governed by the external force field and the mechanical stiffness distribution in the object. We assume that the magnetic particles are well coupled to the surrounding tissues, so the surrounding tissues also move along with the magnetic particles in motion under the magnetic force. We also assume that the amount of nanoparticles circulating the blood vessels near the tissues of interest is relatively small as compared to the ones deposited in the tissues so the effects of circulating nanoparticles are negligible. The tissue mechanics mathematically relates the force and the resultant deformations in terms of mechanical properties. Though living tissues exhibits complex mechanical behavior, they are commonly assumed to be linear, elastic and isotropic material under the consideration of small and brief load application [10-12]. The well known Navier's equation gives the force-displacement relationship for linear, isotropic elastic material following the governing equation of elasticity and Newton's equation of motion [13]. Navier's equation has been widely adopted in ultrasound and MR elastography [11,12] for diagnosis of tumor or cancerous tissues. In this study, we simplify the Navier's equation to obtain the force map using the displacement data. Neglecting the effect of the gravitational force, Navier's equation of the displacement field **u **[m] under the magnetic force field **F**, is [14]:

(2) $$\rho \frac{\partial^{2}u}{\partial t^{2}}=G\nabla^{2}u+\frac{G}{1-2\nu }\nabla \left(\nabla \cdot u\right)+F$$

where *G*, *ν*, and *ρ *are the shear modulus [kPa], the Poisson's ratio, and the density of the medium [kg/m^3^] respectively. Assuming static condition, the equation (2) can be written as:

(3) $$F=-G\nabla^{2}u-\frac{G}{1-2\nu }\nabla \left(\nabla \cdot u\right).$$

This simplified equation implies that we can find the magnetic force field from the displacement field if we know the mechanical stiffness parameters *G *and *v *in advance. Since we usually perform molecular imaging on an interested region inside a living body, we may presume that we know the mechanical stiffness parameters beforehand. Otherwise, an elasticity imaging, such as ultrasound elastography, of the interested region will be needed before applying the magnetic field.

Ultrasound elastography is well known as an efficient approach to measure the displacement of tissues as small as 10 μm [10]. Since ultrasound elastography is capable of displacement measurement only in the axial direction, we have to approximate equation (3) so that it includes only the axial component, i.e., the *z*-component. Since biological tissues are known to be almost incompressible [15], we may presume that the divergence term in equation (3) plays a minor role. Then, Laplacian of the *z*-directional displacement data can give the magnetic force map:

(4) $$F_{z}=-G\left(\frac{\partial^{2}u_{z}}{\partial y^{2}}+\frac{\partial^{2}u_{z}}{\partial z^{2}}\right)$$

In equation (4), we neglect the double derivative in the *x*-direction (elevational direction in ultrasound imaging) only taking account of the *y*- (lateral) and *z*- (axial) directions. In the results section we will observe the effects of the omission of divergence term on the magnetic force map.

### Magnetic coil system design

Uniform magnetic field can be generated using either magnetic coils or permanent magnets. We consider magnetic coils here since they are more convenient to use for clinical applications. Helmholtz coils are widely used to produce uniform magnetic field which consists of two parallel circular coils with same currents flow. The magnetic field produced by a Helmholtz coil oriented along the *z*-axis is given by:

(5) $$B_{z}=\frac{\mu_{0}n_{1}I_{1}a^{2}}{2{\left[{\left(\frac{d}{2}-z\right)}^{2}+a^{2}\right]}^{3/2}}+\frac{\mu_{0}n_{1}I_{1}a^{2}}{2{\left[{\left(\frac{d}{2}+z\right)}^{2}+a^{2}\right]}^{3/2}}$$

where *a *is the coil radius [m], *d *the interspacing [m] between the upper and lower coils, *n*_1 _the number of coil turns, *I*_1 _the coil current, *μ*_o _the magnetic permeability [wb/(A·m)] in free space. A Helmholtz coil makes most uniform magnetic field at the center of the coil when the coil radius *a *and the coil interspacing *d *satisfies *d *= *a*. Then, the magnetic field at the center of the Helmholtz coil is given as *B_z_*(0, 0, 0) = 0.716 *μ*_0_*n*_1_*I*_1_/*a*.

**Figure 1:**
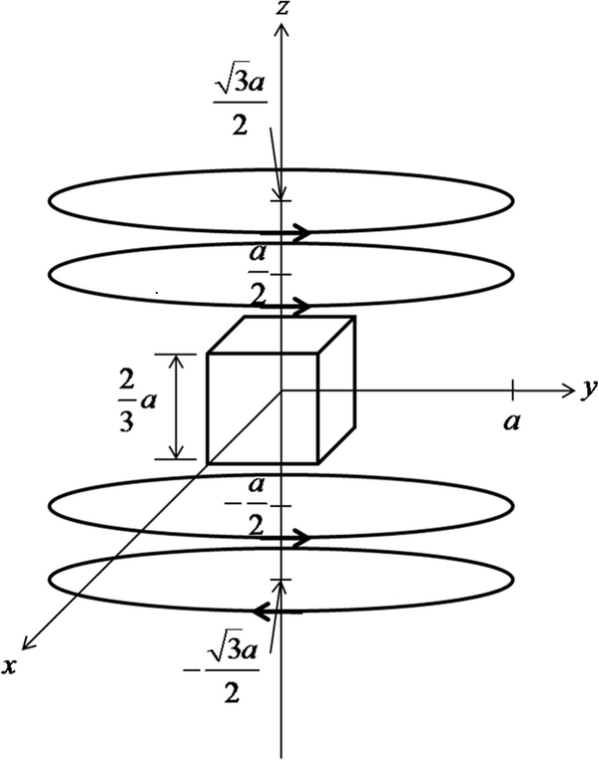
**The magnetic coil configuration**. The Helmholtz/Maxwell coil pair with the cubic imaging region.

We propose to use a Maxwell coil to make linear magnetic field gradient. A Maxwell gradient coil consists of two parallel circular coils along which currents with the same magnitude flow in opposite direction. The magnetic field gradient [Tesla/m] of a Maxwell coil oriented along the *z*-axis is given as:

(6) $$GR_{z}=\frac{dB_{z}}{dz}=\frac{3\mu_{0}n_{2}I_{2}a^{2}}{2}\left\{\frac{\frac{d}{2}-z}{{\left[{\left(\frac{d}{2}-z\right)}^{2}+a^{2}\right]}^{5/2}}+\frac{\frac{d}{2}+z}{{\left[{\left(\frac{d}{2}+z\right)}^{2}+a^{2}\right]}^{5/2}}\right\}$$

where *a *is the coil radius, *d *the interspacing between the upper and lower coils, *n*_2 _the number of coil turns, *I*_2 _the coil current. A Maxwell coil makes most linear gradient field when the coil radius *a *and the coil interspacing *d *satisfy $d=\sqrt{3}a$. Then, the gradient of the magnetic field at the center of the coil is simply given as *GR_z_*(0, 0, 0) = 0.641 *μ*_0_*n*_2_*I*_2_/*a*^2^. Figure 1 shows the Helmholtz and Maxwell coil pair with same coil diameters.

**Figure 2:**
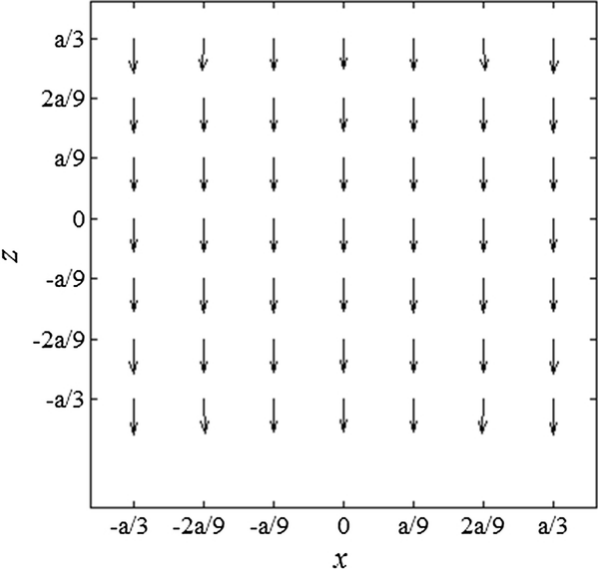
**Magnetic force vectors produced by the coil system**. Normalized magnetic force vectors at the x = 0 plane generated by the pair of Helmholtz and Maxwell coils.

**Figure 3:**
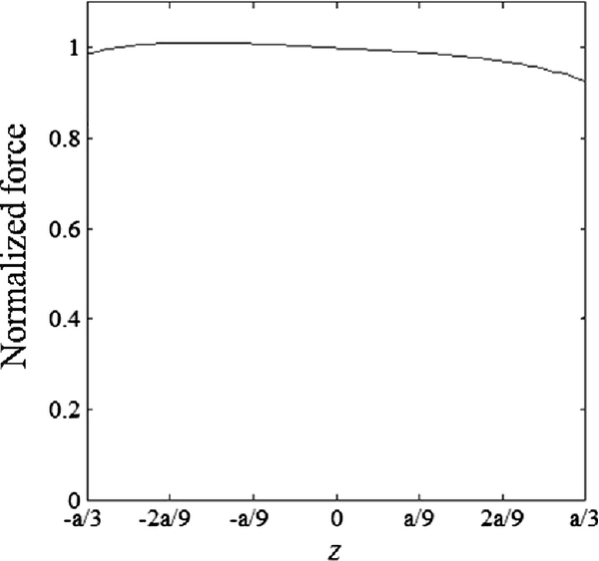
**Magnetic force along the axial direction**. Normalized magnetic forces along the *z*-axis generated by a pair of Helmholtz and Maxwell coils.

To estimate the coil currents needed to induce measurable displacements, we arbitrarily set the imaging volume in the magnetic coils to be (2*a*/3)^3 ^as shown in Figure 1. The normalized magnetic force distribution on the plane of *x *= 0 in the middle of the imaging region is depicted in Figure 2, when the Helmholtz coil current is 10 times bigger than the Maxwell coil current. We assumed the numbers of coil turns equal for both Helmholtz and Maxwell coils. Since the magnetic force is cylindrically symmetric about the *z*-axis, the magnetic force distribution on other planes on the *z*-axis will be the same as Figure 2. Figure 3 shows the normalized magnetic forces generated by the coil pair along the z-axis. The magnetic force field is quite uniform along the axial direction, which is essential for quantitative mapping of the magnetic particle density.

### FEM analysis of magnetic nanoparticle density mapping

With FEM analysis, we have first verified validness of equation (4) where we conveniently neglected the divergence term. We have also established FEM models to estimate the magnetic forces strong enough to make the measurable displacement. In ultrasound elastography, it is well known that the optimum strain level to be measured by ultrasound imaging is about 0.1 ~ 1.0% considering the decorrelation and SNR level of the ultrasound RF signals [16,17].

**Figure 4:**
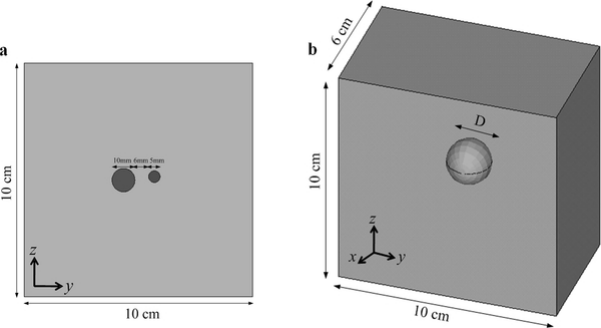
**The FEM models for total force and displacement calculations**. The 2D (**a**) and 3D (**b**) FEM model with cylindrical and spherical inclusions, respectively. We apply uniform force to the inclusions only. The 2D model is for simulating the displacement field and the 3D model for estimating the total force required to induce measurable displacement considering the inclusion diameter D.

Figure 4a, b show the 2D and 3D FEM models, respectively, used in the FEM analysis. The 2D model has the size of 10 cm × 10 cm and it has one or two cylindrical inclusions, and the 3D model has the size of 10 cm × 10 cm × 6 cm and it has a spherical inclusion of diameter *D *[m] at the center. The inclusions mimic the magnetic-particle-containing regions. The 2D model is for calculating the displacement map to be used for derivation of the magnetic force map, and the 3D model is for estimating the total force to induce the measurable displacement level considering the inclusion with varying diameter *D*. The Young's modulus and the Poisson ratio of the models are set to 5 ~ 30 kPa and 0.495, respectively with the density of 1000 kg/m^3^, to mimic normal breast tissues [18,19]. In the models, the inclusions and the background medium have the same mechanical stiffness parameters. We applied uniform body force inside the inclusions, i.e., the same force at every node inside the inclusions, and zero forces in the background. Therefore, the inclusions represent the regions that have uniform magnetic particle density in them. We applied the force along the *z*-direction from the bottom to the top of the model. We calculated displacement fields using a commercial FEM package, ANSYS (ANSYS, Inc., Canonsburg, U.S.A.).

## Results

**Figure 5:**
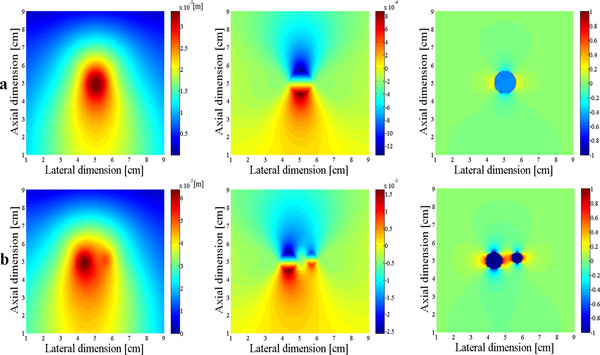
**The displacement, strain, and Laplacian maps**. The displacement (left), strain (middle), and Laplacian of displacement (right) induced by the uniform force applied to the 2D models with a single inclusion (**a**) and double inclusions (**b**).

Figure 5a, b show the displacement (left), strain (middle) and force (right) maps calculated from the 2D model with a single inclusion and two inclusions, respectively, when the desired peak strain of about 0.1% has been established in the inclusion. All the figures show the *z*-directional components, i.e., the axial components. In calculating the force map, we used equation (4) excluding the divergence term. At the single inclusion model, the inclusion has the diameter of 10 mm. At the two inclusion model, the bigger inclusion has the diameter of 10 mm while the smaller one has the diameter of 5 mm. We observe that in spite of presence of halo-like artifacts around the inclusion, the force maps derived by taking Laplacian of the displacement well represent the original uniform force in the inclusion.

**Figure 6:**
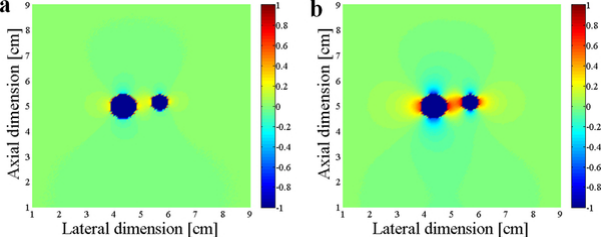
**The normalized force maps**. The normalized force maps obtained with (**a**) and without (**b**) taking account of the divergence term.

**Figure 7:**
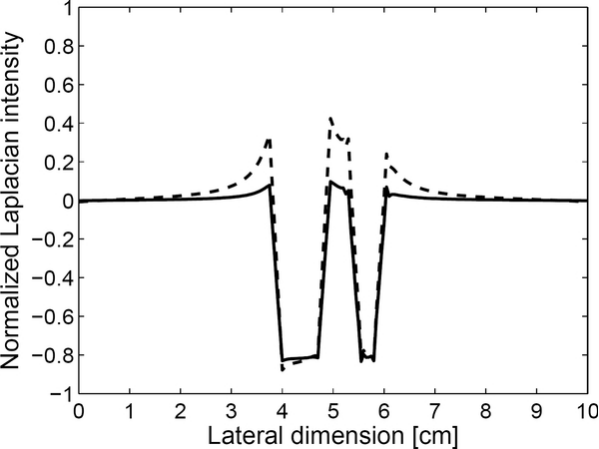
**Effects of neglecting the divergence terms**. The cut views of the force maps obtained with (solid line) and without (dashed line) taking account of the divergence term.

**Figure 8:**
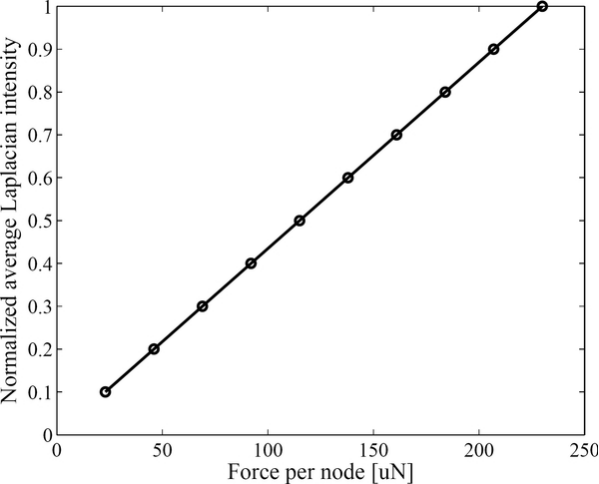
**Relationship between the average Laplacian intensity and the input force**. The average Laplacian intensity at the inclusion with respect to the input force applied to the inclusion in the FEM model.

Figure 6a, b compares the normalized force maps obtained with and without the divergence term, respectively. In calculating the divergence term, we considered all the three components of the displacement. As can be noticed from Figure 6, the halo-like artifacts are greatly reduced by taking into account the divergence term. The divergence term, often considered as pressure gradient in continuum mechanics, also acts as a force term in Navier's equation. We compare the cut views of the force maps passing through the inclusions in Figure 7. Without considering the divergence term, we have some overshoots around the inclusion boundary. Figure 8 shows the relation between the input force applied to the inclusion and the output average force intensity observed at the force maps that have been calculated without considering the divergence term. In spite of neglecting the divergence term, the input force and the observed output force show good linear relationship.

**Figure 9:**
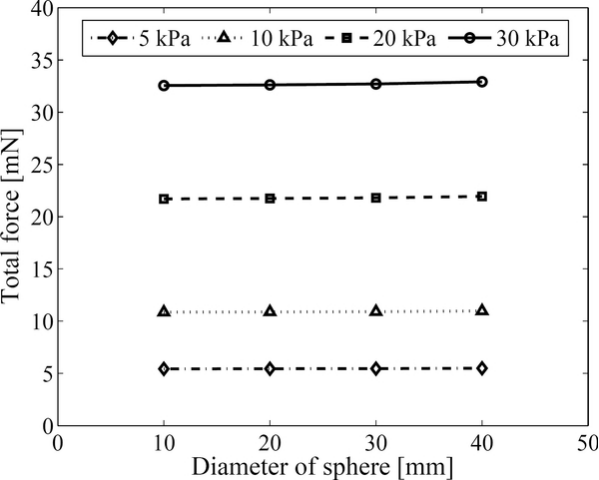
**The total magnetic force to induce 10 μm peak displacement**. The total forces required to induce 10 μm peak displacement in the spherical inclusions with different level of stiffness.

The total force required to induce the measurable displacement level of 10 μm at the inclusion is calculated using FEM analysis on the 3D model. We set the diameter of the spherical inclusion to 10 mm ~ 40 mm and the Young's modulus of the medium to 5 ~ 30 kPa mimicking soft tissues. The total forces to induce peak displacement of 10 μm are summarized in Figure 9. We observe that the total force is not strongly dependent on the inclusion size and harder tissue needs stronger force to induce same level of displacement.

**Figure 10:**
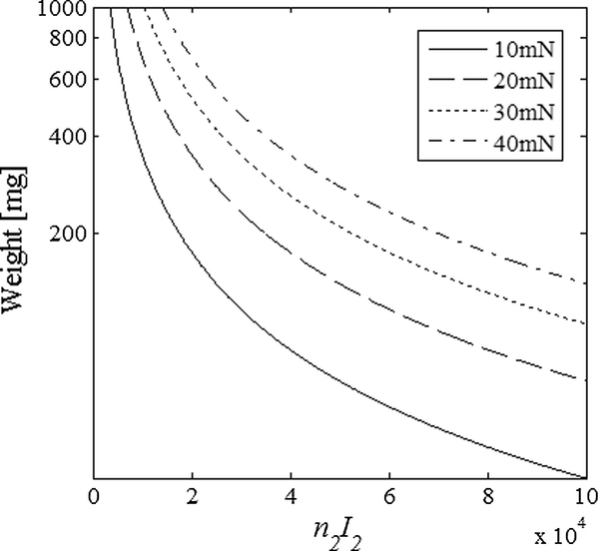
**Maxwell coil currents**. The Maxwell coil currents and the nanoparticle weights to induce measurable displacement of 10 μm in soft tissues with respect to the required total force. The diameter of the Maxwell coil is 15 cm.

To estimate the coil currents required to induce the measurable displacement, we set the coil diameter to 15 cm that would be suitable for human breast imaging. Most of nanoparticles become saturated at the magnetic field of 0.2 ~ 0.5 Tesla at the ambient temperature and the saturated magnetization is about 20-40 Am^2^/kg [20]. If we magnetize the nanoparticles at the magnetic field of 0.4 Tesla with the Helmholtz coil, then the coil current times the number of turns, *n*_1_*I*_1_, is about 3.34 × 10^4^A. If we assume the saturated magnetization be 20 Am^2^/kg, the magnetic force at the centre of the coil set will be Fz(0, 0, 0) = 12.8 *w μ*_0_*n*_2_*I*_2_/*a*^2 ^where *w *is the nanoparticle weight. Nanoparticle weight at the region of interest will be largely dependent on the total amount of the nanoparticles administered to the living body and how much portion of the nanoparticles is delivered to the region of the interest. Assuming the maximum particle weight of 1 g at the region of interest, the calculated Maxwell coil currents are shown in Figure 10 that make 10 ~ 40 mN forces.

## Discussion

Navier's equation tells that we can derive magnetic force maps from the displacement maps. To get precise force maps, we need to know all the three components of the displacement vector field. But, we think measuring all the three components is not feasible whatever types of imaging modalities we use considering their physical limitations like spatial resolution and scan time. Ultrasound imaging is a well known and most precise tool for measuring the axial displacement at tissues as thick as 10 cm. Optical coherence tomography can measure the axial displacement with much higher precision than ultrasound imaging, by an order of tens of nm, but it can measure axial displacements at only shallow region no deeper than several mm [21]. If we use the axial displacement component only, we can have the magnetic force map with some halo-like artifacts around the boundary of the magnetic-particle-containing region. Despite the halo-like artifacts in the force maps, it has been found that the average intensity in the force maps is linearly proportional to the magnetic particle density. This implies that the magnetic force maps may give valuable information necessary for quantification of magnetic particle deliveries in a living body.

We need to consider some technical challenges for practical use of the proposed method. We need a coil driving system with high output current capacity along with proper coil cooling mechanism. Coil vibration during the coil driving may cause erroneous displacement in the imaging region if the imaging region is not well mechanically isolated from the coil system. But considering the recent report on the optical measurement of displacement with precision of tens of nm, induced by magnetic nanoparticles under time-varying magnetic field [21], it seems that magnetic coil vibration can be decoupled to a sufficient level.

## Conclusions

In conclusion, magnetic force maps or magnetic nanoparticle density maps can be derived from the displacement induced by the external magnetic field. Even though the magnetic force maps have halo-like artifacts around the magnetic-particle-containing regions, we think the proposed method may find applications in real-time molecular imaging studies with an ultrasound scanner.

## Abbreviations

**F**: magnetic force [N]; **m**: magnetic dipole moment [A·m^2^]; **B**: magnetic field density [Tesla]; **a**: unit vector; **u**: displacement vector [m]; **H**: magnetic field intensity [A/m]; *χ*: magnetic susceptibility; *V*: volume [m^3^]; **M**: effective magnetization [A/m]; *ρ*: material density [kg/m^3^]; *G*: shear modulus [kPa); *ν*: Poisson's ratio; *t*: time [s]; *μ*: magnetic permeability [wb/(A·m)]; *a*: coil radius [m]; *d*: spacing between upper and lower coils [m]; *n*: number of coil turns; *I*: coil current [A]; *GR*: gradient field [Tesla/m]; Subscripts: 0: in free space; *k*: nanoparticle index; *z*: axial direction; 1: Helmholtz coil; 2: Maxwell coil.

## Competing interests

The authors declare that they have no competing interests.

## Authors' contributions

ABMAH has carried out mathematical modeling and computer simulation as well as drafted the manuscript. MHC has guided ABMAH in developing the numerical programs. SYL has suggested the theoretical concepts and finalized the manuscript. All authors have read and approved the final manuscript.
